# Characterization of New Otic Enhancers of the Pou3f4 Gene Reveal Distinct Signaling Pathway Regulation and Spatio-Temporal Patterns

**DOI:** 10.1371/journal.pone.0015907

**Published:** 2010-12-31

**Authors:** Àlex Robert-Moreno, Silvia Naranjo, Elisa de la Calle-Mustienes, José Luis Gómez-Skarmeta, Berta Alsina

**Affiliations:** 1 Department of Experimental and Health Sciences, Universitat Pompeu Fabra/Parc de Recerca Biomèdica de Barcelona, Barcelona, Spain; 2 Centro Andaluz de Biología del Desarrollo, CSIC/Universidad Pablo de Olavide, Sevilla, Spain; Texas A&M University, United States of America

## Abstract

POU3F4 is a member of the POU-homedomain transcription factor family with a prominent role in inner ear development. Mutations in the human *POU3F4* coding unit leads to X-linked deafness type 3 (DFN3), characterized by conductive hearing loss and progressive sensorineural deafness. Microdeletions found 1 Mb 5′ upstream of the coding region also displayed the same phenotype, suggesting that cis-regulatory elements might be present in that region. Indeed, we and others have recently identified several enhancers at the 1 Mb 5′ upstream interval of the *pou3f4* locus. Here we characterize the spatio-temporal patterns of these regulatory elements in zebrafish transgenic lines. We show that the most distal enhancer (HCNR 81675) is activated earlier and drives GFP reporter expression initially to a broad ear domain to progressively restrict to the sensory patches. The proximal enhancer (HCNR 82478) is switched later during development and promotes expression, among in other tissues, in sensory patches from its onset. The third enhancer (HCNR 81728) is also active at later stages in the otic mesenchyme and in the otic epithelium. We also characterize the signaling pathways regulating these enhancers. While HCNR 81675 is regulated by very early signals of retinoic acid, HCNR 82478 is regulated by Fgf activity at a later stage and the HCNR 81728 enhancer is under the control of Hh signaling. Finally, we show that Sox2 and Pax2 transcription factors are bound to HCNR 81675 genomic region during otic development and specific mutations to these transcription factor binding sites abrogates HCNR 81675 enhancer activity. Altogether, our results suggest that *pou3f4* expression in inner ear might be under the control of distinct regulatory elements that fine-tune the spatio-temporal activity of this gene and provides novel data on the signaling mechanisms controlling *pou3f4* function.

## Introduction

The inner ear of vertebrates is one of the most complex sensory organs of the head and host two senses, the sense of hearing and the sense of balance. From an ectodermal layer adjacent to the hindbrain, the otic placode, a spheroid organ is generated by invagination/cavitation followed by a series of developmental processes such as patterning, cell diversification and morphogenesis. In the ventral portion of the inner ear, the cochlea or auditory organ emerges as an outpocketing of the otic vesicle, while in the dorsal portion of the otic vesicle the vestibular organs, semicircular canals and endolymphatic duct are developed. In each sensory organ, the main functional unit is composed by the hair-cells, the supporting cells and the sensory neurons that connect the hair-cells to the central nervous system [1], [2]. The integration of signals in the inner ear from the surrounding tissues is essential for its proper development. In recent years, a large number of genes have been disclosed to participate in the formation of the ear and control gene activity. Yet, how those interact and are spatio-temporally regulated is still poorly understood. Highly conserved non-coding regions (HCNR) have been revealed and proposed to contain key cis-regulatory elements [3], [4]. Emergent characteristics of these sequences are their evolutionary conservation, their location in the genome (either upstream or downstream and even in introns from the gene that regulate), their clustering around transcription factors and their contributions to disease when mutated [5]–[7]. To date, very few regulatory regions controlling inner ear gene transcription have been identified so far. Recently, a regulatory region of the *Dlx5-Dlx6* genes was found by the study of five affected members displaying hearing loss and craniofacial defects. The affected individuals shared a deletion of 5,115 bp. Bioinformatic analysis of this sequence indicated the presence of several HCNR, which in a transgenic mouse reporter assay, drove expression in the inner ear and developing bones [8].

The POU proteins are transcription factors that bind to DNA through their POU-specific and POU-homeodomain regions and play essential roles during development. Several members of the POU family are expressed in the inner ear. The gene *POU4F3* (*Brn3c*) is specifically expressed in hair-cells and mutations in *POU4F3* causes DFNA15, an autosomal dominant form of progressive hearing loss [9]. Mutations in another member of the POU family, the *POU3F4* (Brn4) causes deafness type 3 (DFN3), characterized by a conductive hearing loss that results from stapes fixation and progressive sensorineural deafness [10]. The human gene *POU3F4* is located in the X chromosome (Xql3-q22) being one of the most frequent causes of X-linked deafness. In rats, the *Pou3f4* gene is expressed during embryonic development in the brain, the neural tube, and the otic capsule at E15.5 and E17.5 days [11]. In mice, mutations of the homologous gene cause similar defects as in humans. Loss of the tissues derived from the otic mesenchyme was reported, as well as a shortening of the cochlea suggesting that *Pou3f4* might be required for epithelial-mesenchymal interactions taking place during development [12], [13]. In humans, in addition to mutations in the coding region, a hotspot 920 Kb 5′ upstream of the *POU3F4* gene was identified where several microdeletions also caused DFN3 phenotype, suggesting that regulatory regions were present in that region [10], [14]–[16]. Recently, using comparative genomics and transgenic assays in different model systems, a *POU3F4* enhancer within a HCNR (HCNR 81728) was described to induce reporter expression in the otic mesenchyme. This enhancer lay within the smallest microdeletions shown to cause DFN3 [17], [18]. However, since not all microdeletions affect this enhancer [16], [19], it was hypothesized that other enhancers might be present in the hotspot region. Reported in Naranjo et al. (2010) [18], we have identified two additional enhancers at HCNR located within the 1 Mb 5′ upstream of the *Xenopus* coding region, at position 970 Kb (HCNR 81675) and 170 Kb (HCNR 82478) of the *pou3f4* coding unit that present otic vesicle enhancer activity in a zebrafish transgenesis assay. Here, we have analyzed the spatio-temporal activity of these enhancers, as well as the signaling pathways that initiate their activity. We found that the HCNRs display distinct temporal patterns of activation and; while HCNR 81675 is regulated by the retinoic acid (RA) signaling, the HCNR 82478 is regulated by the Fgf pathway and the HCNR 81728 enhancer is under the control of Hh. Finally we present direct evidence that the distal enhancer is bound *in vivo* by Sox2 and Pax2 transcription factors. Altogether, these data suggest that the regulatory apparatus of the *pou3f4* is multiple, complex and integrate distinct developmental inputs.

## Materials and Methods

### Ethics Statement

Zebrafish transgenic fishes have been maintained at the PRBB Animal Facility. Our Animal Facility in accordance with national and European regulations is registered as animal research center with the number B9900073. Veterinary welfare supervision and daily water check-ups are conducted (dissolved oxygen, conductivity, pH, ammonia, nitrites, nitrates, alkalinity and hardness -Kh and Gh-, among other parameters) to ensure the animals good health status. Temperature, humidity, light intensity and noise control in the room are strictly monitorized to guarantee animal welfare. Zebrafish embryos have been sacrificed after being anesthetized with 0.016% tricaine when necessary. The experimental zebrafish procedures have been performed following the protocols (CEEA-PRBB ref JMC-07-1001-CPC and MM-08-1108BAE) approved by the Ethical Committee for Animal Research (CEEA) from Barcelona Biomedical Research Park (PRBB) according to the European Union regulations.

### Generation of transgenic zebrafish lines

*pou3f4* HCNR 81675 and HCNR 82478 GFP zebrafish lines were obtained as described in [18]. Briefly, both HCNRs were selected based on high sequence conservation with human genome by using VISTA browser (http://pipeline.lbl.gov/cgi-bin/gateway2). Subsequently genome fragments were amplified by PCR from *Xenopus tropicalis*, cloned into the PCR8/GW/TOPO vector and stable zebrafish lines generated by the Tol2 transgenesis system.

### Transgenic GFP detection

*pou3f4* HCNR 81675 and HCNR 82478 GFP embryos were staged according to morphology and somite pair number. For life GFP imaging, tricaine-anesthesized embryos were mounted in slides with glycerol and images were taken under the microscope.

### Whole-mount in-situ hybridization

Whole-mount in-situ hybridization (WISH) was performed according to standard protocols [20]. Briefly, dechorionated zebrafish embryos were fixed in 4% paraformaldehyde overnight at 4°C and dehydrated in methanol series, rehydrated again and treated with 10 µg/ml proteinase K (Sigma) for 10 minutes. Digoxigenin-labelled probes were hybridized overnight at 58°C, detected using sheep anti-digoxigenin-AP antibody at 1∶2000 dilution (Roche) and developed with NBT/BCIP (Roche). Embryos were either used for imaging or embedded in tissue-tek OCT (Sakura) for sectioning in a Leica CM1510-1 cryostat at 12 µm.

### Inhibitor treatment assay

HCNR 81675 and HCNR 82478 GFP transgenic embryos were chorion punctured and incubated with different pharmacological inhibitors in system water, from 5.5, 7.5 or 9.5 hours post-fertilization (hpf) stage until 18–20 hpf or 36–40 hpf respectively. The battery of inhibitors included: 30 and 50 µM SU5402 (Calbiochem) to inhibit Fgf signaling, 100 µM DAPT (Calbiochem) to inhibit Notch signaling, 20 µM DEAB (Sigma) to block RA synthesis, 30 µM Dorsomorphin (Biomol) to inhibit Bmp signaling and 45 µM cyclopamine A (CyA) to inhibit Hh signaling. DMSO diluted at 1/200 in system water or EtOH 95% was used as the carrier control treatment.

### Immunostaining

For immunostaining after WISH, *pou3f4* HCNR 81675 developed embryos were frozen in tissue-tek OCT and sectioned (12 µm). Slides were fixed in −20°C methanol for 10 minutes and blocked-permeabilized in 10% fetal bovine serum (FBS), 3% bovine seric albumin (BSA) (Sigma) in 0.1% PBT (0.1% tween-20) for 90 minutes at room temperature. Slides were stained with rabbit anti-GFP (Takara) at 1∶400 overnight at 4°C and donkey anti-rabbit Alexa 488 (Molecular Probes) at 1∶400 for 90 minutes at room temperature, both in 0.1% PBT, 10% FBS, 3% BSA. Sections were mounted in mowiol for imaging. For double immunostaining, transgenic GFP was able to be detected after all the processing, thus *pou3f4* HCNR 81675 embryos were sectioned, blocked-permeabilized and stained with mouse anti acetylated-tubulin (Sigma T6793) or rabbit anti-Pax2 (Zymed laboratories ref.71-6000) at 1∶400 overnight at 4°C and goat anti-mouse Alexa 546 or goat anti-rabbit Alexa 594 (Molecular Probes) at 1∶400 for 90 minutes at room temperature. Nuclei were stained with 4′6-diamidino-2-phenylindole (DAPI, Molecular Probes) for 5 minutes and mounted in mowiol.

### FM® 4-64FX staining

Alive 3 day-old *pou3f4* HCNR 81675 embryos were injected into the otic vesicle with a micromanipulator with the FM® 4-64FX reagent (Invitrogen) at 1∶5 dilution in water, and then left in system water for 15 minutes, fixed in 4% paraformaldehyde (Sigma) and frozen in tissue-tek OCT (Sakura) for sectioning and imaging.

### Transcription factor binding site (TFBs) prediction and site-directed mutagenesis

In order to predict conserved TFB sites in the *pou3f4* HCNR 81675 sequence, human and *Xenopus* sequences were analysed in rVISTA 2.0 (http://rvista.dcode.org/) that predicts evolutionarily conserved transcription factor binding sites. Moreover, the sequence of *Xenopus* was also analysed by Transfac 7.0 in the Gene Regulation portal (http://www.gene-regulation.com/index.html). Pax2 and Sox2 binding sites found in the *pou3f4* HCNR 81675 were subjected to site-directed mutagenesis. Based on the Transfac motif and literature research, we mutated the core nucleotides of the Pax2 and Sox2 binding sites by designing primers that contained the Pax2 binding core GTGAATAG mutated into TCAAACAT or the Sox2 binding core ACAAAA mutated into GTGCTC. Mutant primers were used to introduce these point mutations into the pCR8/GW/TOPO TA® - HCNR 81675 construct using the Quik Change® XL Site-Directed Mutagenesis Kit (Stratagene). Transgenic zebrafish containing either Pax2 or Sox2 mutant binding sites as well as the double mutant for Pax2 and Sox2 motifs were generated using the ZED vector and Tol2 transposon/transposase transgenesis method [21] and F1 embryos were analyzed for the presence of inner ear GFP.

### Chromatin ImmunoPrecipitation (ChIP) assay

Otic vesicle from stage 24 and stage 30 *Xenopus* embryos were dissected and used for Chromatin immunoprecipitation assay. ChIP analysis was performed with minor modifications as described previously [22]. Chromatin was crosslinked with formaldehyde (Merck), sheared into 200–500 base pairs fragments by sonication with a Bioruptor Diagenode sonicator (medium speed, 8 minutes), incubated overnight with rabbit anti-Pax2 (Zymed laboratories) and rabbit anti-Sox2 (Abcam, Ab15830) antibodies and precipitated with protein G/A-Sepharose (GE Healthcare). DNA-protein complexes were decrosslinked and immunoprecipitated chromatin was used for quantitative PCR performed using SYBR Green I Master kit in a LightCycler480 system (Roche). PCR primers for the Pax2 and Sox2 binding sites in the *Xenopus pou3f4* HCNR 81675 were designed (Sox2 forward TTCCAGTCTTTTCTTTTCCAAAGCT, reverse TTTGCCTTTGGGCGTAATTT; Pax2 forward CAGCATCCATTTAATTCATCAA

ACA, reverse TGAAGTTTCTCTCTTCTGCAACTCTT), whereas primers for a CNR in the *Xenopus haemoglobin-2* locus with no predicted Pax2 and Sox2 binding sites according to rVISTA and Transfac databases were used as negative control (*Xenopus* scaffold_357:988236-988368; forward TCTGCTCTCTTGTAGCTGCTGTCT; reverse ACTTGTCCCAGGCAGCTTGT).

### Image acquisition

Pictures were acquired in a Leica DRM microscope using a Leica DFC300 FX camera and the Leica IM50 software. Adobe Photoshop CS2 software was used for photograph editing.

## Results

### *pou3f4* inner ear enhancers activate gene expression at different developmental stages

**Figure 1:**
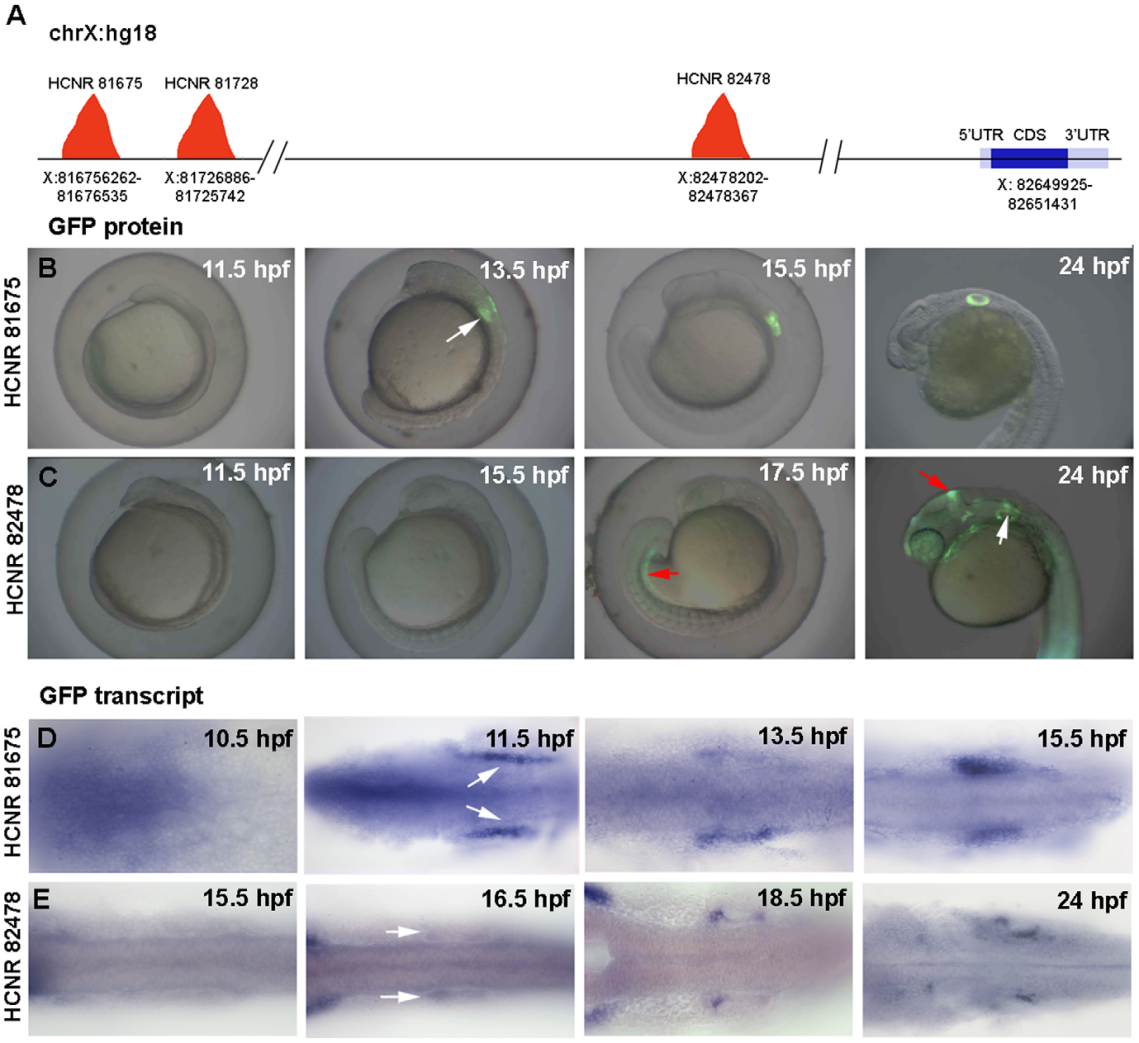
Temporal expression pattern of GFP driven by *pou3f4* HCNR 81675 and HCRN 82478 enhancers. (A) Schematic representation of the *POU3F4* locus in the human chromosome X (hg18 alignment) showing the position of the different inner ear enhancers relative to the *POU3F4* coding sequence. (B–C) Onset of GFP protein expression in HCNR 81675 (B) and HCNR 82478 (C) transgenic embryos. Expression in the otic territory occurs at 13.5 hpf in HCNR 81675 and at 18.5 hpf in HCNR 82478 zebrafish embryos (white arrows), GFP in mesonephros and midbrain-hindbrain boundary (red arrows) is also detected in HCNR 82478 embryos. (D–E) Dorsal views of HCNR 81675 (D) and HCNR 82478 (E) transgenic embryos assayed by in-situ hybridization for GFP mRNA expression. In both cases GFP mRNA was detected in the otic field 2 hours before GFP protein was found (B–C). Orientation of the embryos is anterior (left) to posterior (right).

We have recently used comparative genomics and transgenic assays in *Xenopus* and zebrafish to identify several highly conserved non-coding regions (HCNR) in the 1 Mb 5′ upstream region of the *pou3f4* transcription start site with enhancer activity in the developing inner ear [18]. Here we use the same nomenclature used in our previous work report. Thus, each enhancer is named by their position in the human chromosome X (hg18 version) in kilobases. HCNR 81675, 81728 and 82478 are located, respectively, at 970, 922 and 70 Kb apart from the *pou3f4* transcriptional start site (Figure 1A). We first determined the onset of expression of each enhancer in stable zebrafish transgenic lines harboring GFP under the control of each of these cis-regulatory regions. In this and in our previous work [18], we observed that GFP driven by the enhancer at HCNR 81728 became clearly visible at 72 hpf (data not shown). Since we are particularly interested in early patterning events during inner ear development, we have concentrated in the other two enhancers, HCNR 81675 and HCNR 82478, which activate transcription much earlier. To that end, embryos were assayed for GFP expression every hour from 10.5 hours post-fertilization (hpf) to 18.5 hpf, and then again at 24 hpf and 36 hpf. Expression in the inner ear was first detected in HCNR 81675 embryos of 13.5 hpf (Figure 1B), whereas for HCNR 82478 otic GFP was not found earlier than 18.5 hpf. Before initiating GFP expression in the inner ear, the enhancer was active in the mesonephros at 17.5 hpf (Figure 1C). The HCNR 82478 also droved expression in the midbrain-hindbrain boundary at 18.5 hpf and to the spinal cord at 24 hpf (Figure 1C).

To check at which stage, both enhancers are functional at the transcriptional level, whole-mount in situ hybridization (WISH) to detect GFP mRNA was performed. WISH in the same stage embryos revealed that, in both *pou3f4* enhancers, GFP mRNA transcription starts two hours before the GFP protein is detectable, at 11.5 hpf and 16.5 hpf respectively (Figure 1D and E). Thus, at the mRNA and at the protein level these results indicate that both enhancers are functional and regulate reporter gene expression to the otic vesicle (among other tissues in the case of HCNR 82478) but they activate the expression at different developmental stages.

### Early activated *pou3f4* enhancers promote expression in inner ear sensory patches

**Figure 2:**
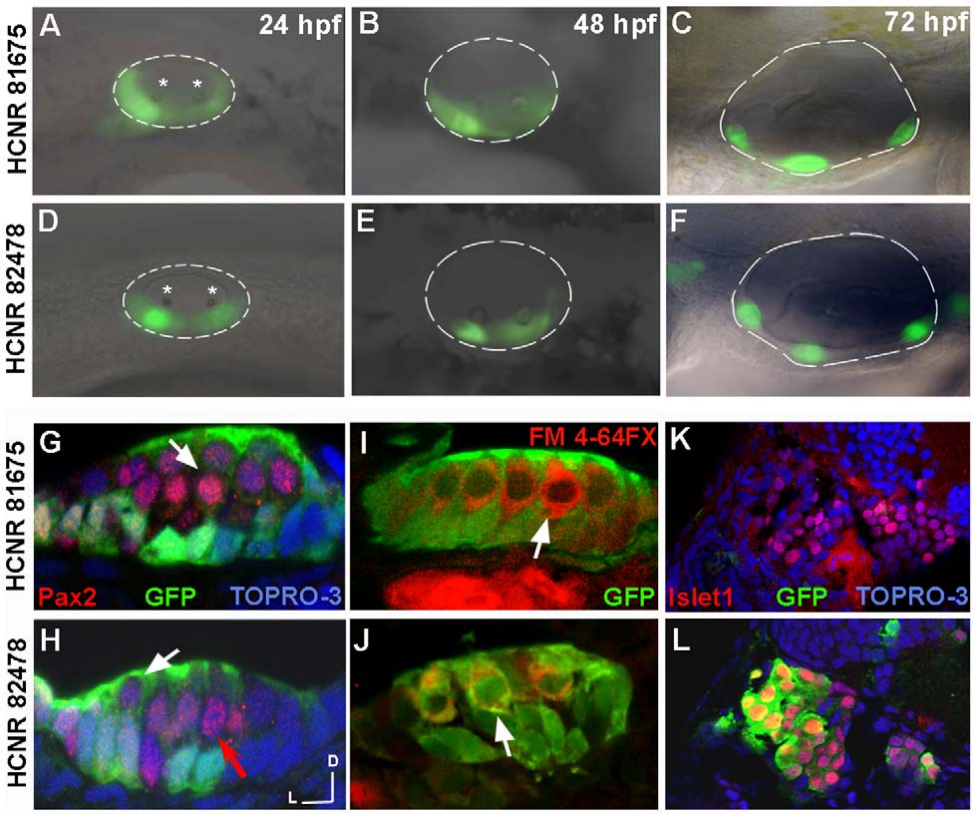
Spatial-temporal expression pattern of *pou3f4* enhancers in the inner ear. (A–F) Lateral views of inner ears from zebrafish transgenic embryos for HCNR 81675 (A–C) and HCNR 82478 (D–F) enhancers analysed from 24 hpf to 72 hpf. In HCNR 81675 embryos at 24 hpf, GFP is observed in two broad domains comprising the sensory territories as observed by the otolith deposition (stars) (B). In HCNR 82478, GFP is already restricted to the anterior and posterior sensory macula from its onset as observed by GFP fluorescence relative to the otolith position (star). (C and F) GFP is found in the three sensory crista in 3-day old embryos in both transgenic zebrafish lines. Orientation is anterior (left) and dorsal (up). (G and H) Confocal transverse images of inner ear sensory patches immunostained with the anti-Pax2 antibody in 72 hpf embryos. In HCNR 81675 embryos, GFP is found in supporting cells but absent in hair-cells (Pax2 positive cells; pointed by a white arrow) (G). In contrast, HCNR 82478 embryos displayed GFP in supporting cells but also in hair-cells at lower levels (white arrow) whereas other hair-cells where completely devoid of GFP expression (red arrow). (I and J) Transverse confocal images of sensory patches of both enhancer embryos immunostained for GFP after the injection of the hair cell specific labelling marker FM 4-64FX. The same result was obtained in this experiment. (I) GFP is devoid in FM 4-64FX stained hair-cells in HCNR 81675 embryos (white arrow), whereas some hair-cells displayed GFP in HCNR 82478 embryos (J; white arrow). (K and L) Confocal images taken from the transverse section anterior to the first section from the otic vesicle. Co-immunostaining for anti-GFP and anti-islet1 protein reveals that only in HCNR 82478 transgenic embryos GFP is activated in the otic ganglion (L). (G–L) Lateral (left) and dorsal (up).

Since both *pou3f4* early expressed enhancers activate reporter GFP expression at different developmental stages in zebrafish, we next assayed whether the spatial pattern of expression in the inner ear also presented particularities. From 13 hpf to 24 hpf, GFP driven by HCNR 81675 is observed in almost the entire otic placode (see Figure 1B). However, at 24 hpf a lateral view of the otic vesicle revealed that higher expression is concentrated ventrally, in a broad domain that includes the areas of anterior and posterior otolith deposition (Figure 2A, otoliths indicated with a star). Otoliths appear at 24 hpf and are particles of gelatinous matrix and calcium carbonate that are deposited over the hair-cells of the maculae, helping to the sense of gravity and linear acceleration. On the other hand, expression promoted by HCNR 82478 at 24 hpf is already restricted to the sensory domain, as judged by the correspondence with anterior and posterior otolith (Figure 2D). When embryos reach the 3-day old stage, GFP expressions driven by both *pou3f4* HCNRs become restricted to the two sensory maculae and the three sensory cristae associated to the semicircular canals (Figure 2C and F).

Sensory patches are formed by hair-cells and supporting cells. Thus, to determine whether GFP is restricted to any lineage of the otic sensory patches or by contrast expressed in both cell types, we performed staining either with an antibody against Pax2 protein or with FM 4-64FX compound. In 3-day embryos, the Pax2 protein is found only in hair-cells nuclei. On the other hand, the FM 4-64FX compound stains hair-cell cytoplasm by entering through ion channels of the stereocilia. Interestingly, in the case of HCNR 81675 enhancer, GFP staining was observed in supporting cells but excluded from hair-cells labelled with Pax2 or FM 4-64FX compound (shown in red in Figure 2G and I, white arrows). However, in the case of HCNR 82478, GFP staining was observed both in hair-cells and supporting cells (Figure 2H and J; white arrows). GFP was also found in the otic ganglion in HCNR 82478 (but not for HCNR 81675) transgenic fishes as shown by cells co-immunostained with anti-GFP and anti-Islet1 in neuroblasts anterior to the otic vesicle (Figure 2K and L).

### Distinct *pou3f4* inner ear enhancers are under the control of different signaling pathways

**Figure 3:**
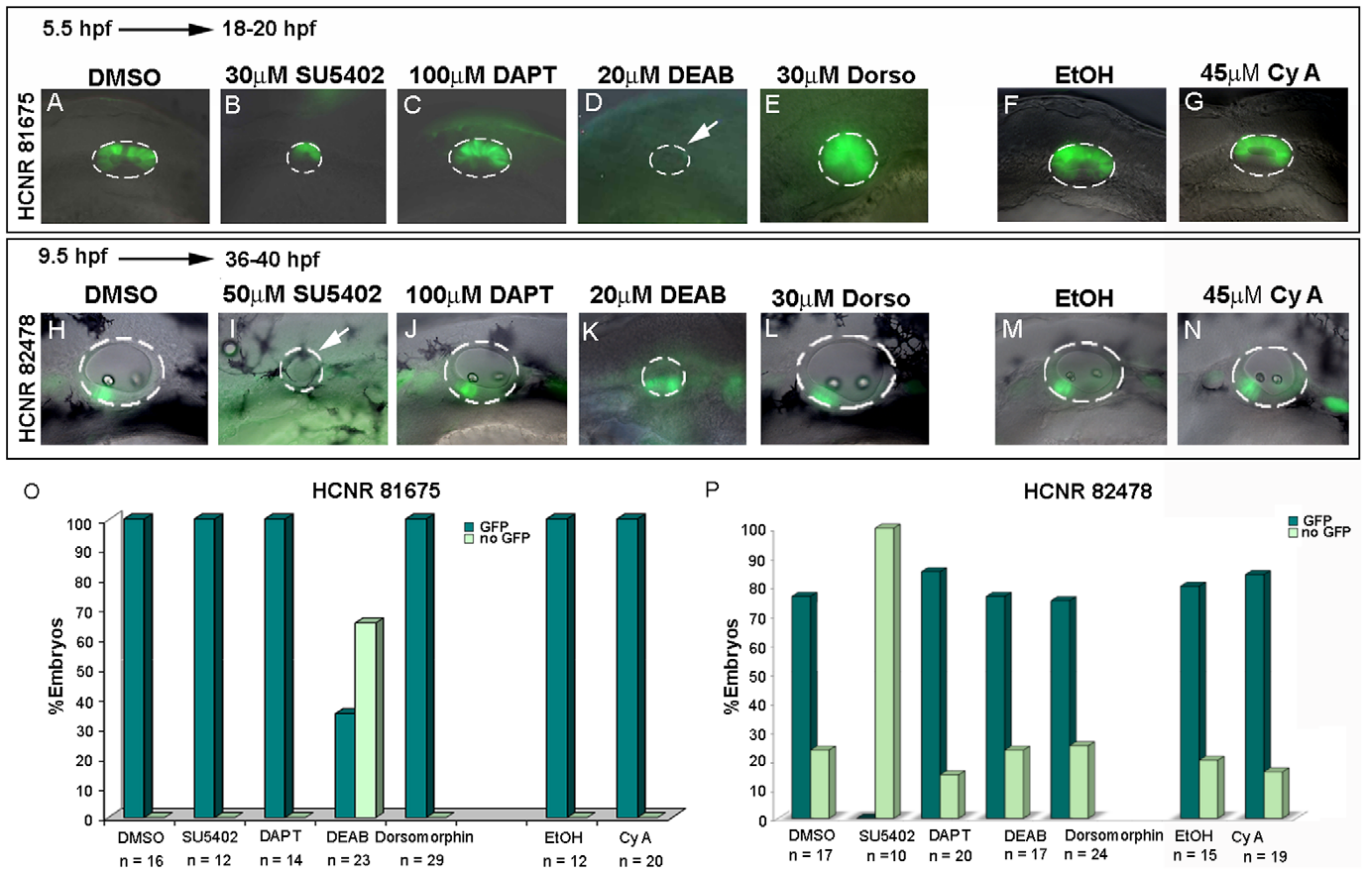
Distinct signaling pathways regulate activation of *pou3f4* HCNR 81675 and HCNR 82478 enhancers. (A–N) Transgenic embryos for both enhancers were treated with different pharmacological inhibitors from 5.5 hpf stage to 18–20 hpf and 7.5 hpf to 36–40 hpf respectively. Lateral view of HCNR 81675 (A–G) 18–20 hpf staged otic vesicles and HCNR 82478 (F–N) otic vesicles of 36–40 hpf embryos. HCNR 81675 activity was abrogated in the presence of RA signaling inhibitor DEAB (compare D to the control treatment with DMSO in A), whereas Fgf signaling inhibition by SU5402 completely disrupted *pou3f4* HCNR 82478 activity (compare I to control treatment in H). Orientation is anterior (left) to posterior (right). (O, P) Graphs representing the percentage of embryos displaying complete inhibition of GFP expression in *pou3f4* HCNR 81675 (O) and HCNR 82478 (P) transgenic embryos after specific signaling pathway blockade. The total number of embryos counted in three independent experiments is represented.

Next we decided to address which signaling pathway/s might control the activation of the different *pou3f4* enhancers *in vivo*. Transgenic embryos for the HCNR 81675 enhancer were treated from different temporal points with a battery of pharmacological inhibitors of distinct signaling pathways. When embryos were treated with inhibitors of the Fgf (SU5402), the RA (DEAB) and Bmp (Dorsomorphin) pathways, smaller otic vesicles were observed since all three pathways play essential roles in otic placode formation [23]–[28]. In contrast, Notch (DAPT) and Hh (CyA) pathways inhibition did not have a major effect on the size of the otic vesicle. Interestingly, the expression of GFP driven by HCNR 81675 enhancer was only lost when RA signaling was abrogated at 5.5 hpf (Figure 3A–G and 3O) but not at later stages such as 7.5 hpf or 9.5 hpf (Figure S1), indicating that the HCNR requires an early RA signal to be induced. Note that at these stages, Fgf was inhibited at a low concentration of SU5402, since at a concentration of 50 µM otic vesicle formation is severely compromised due to the requirement of Fgf in otic induction [26]–[29]. In situ hybridization for Fgf, Notch, RA, Bmp and Hedgehog target genes such as *pea3*, *neuroD*, *krox20*, *msx1* and *ptc1* was done in inhibitor-treated embryos to confirm that each signaling pathway was abrogated at our working concentrations (Figure S2).

A similar experimental procedure was performed for the HCNR 82478 enhancer. We treated the transgenic embryos with the same battery of signaling pathway inhibitors from 5.5 hpf, 7.5 hpf (Figure S3) and 9.5 hpf stage to up to 36–40 hpf (Figure 3H–N and 3P). In this case, embryos were incubated until later stages when strong GFP signal in the otic vesicle is detected. Interestingly, RA signaling blockade did not suppress the activity of HCNR 82478 enhancer but instead GFP expression was lost after Fgf signaling inhibition at all stages tested. These data indicate that both enhancers are regulated independently and are active under the influence of distinct developmental pathways.

**Figure 4:**
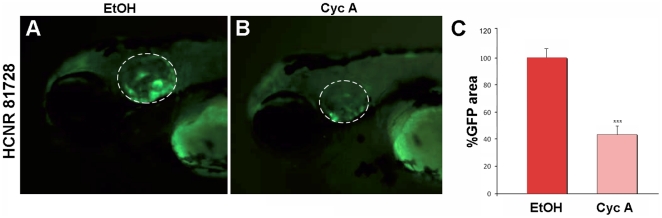
The *POU3F4* HCNR 81728 enhancer is regulated by Hedgehog signaling. (A and B) Lateral view of GFP otic expression in 96 hpf embryos transgenic for the HCNR 81728 enhancer in control (A) and Cyclopamine A treated embryos (B). (C) Percentage of GFP expressing area in otic vesicles from 95% EtOH and Cyclopamine A treated embryos.

In mice, Pou3f4 works in a cooperative manner with Tbx1 transcription factor to control cochlear growth [30] and mesenchymal expression of *Tbx1* has been shown to be under the control of Shh [31]. Thus, we decided to test whether the HCNR 81728 enhancer was also under the control of this pathway in zebrafish transgenic lines. Note that in zebrafish, GFP expression is mainly detected in the otic vesicle, with some expression also at the mesenchyme (Figure 4A and [18]). We found that, in contrast to the other enhancers, the HCNR 81728 enhancer displayed a strong reduction of the area of GFP expression (expressed as % of GFP domain) after blockade of Hh pathway by CyA (Figure 4A–C) suggesting that probably *tbx1* and *pou3f4* share the same regulatory mechanisms.

### GFP driven by the *pou3f4* HCNR 81675 enhancer co-localizes with *pax2a* and *sox2* mRNA

**Figure 5:**
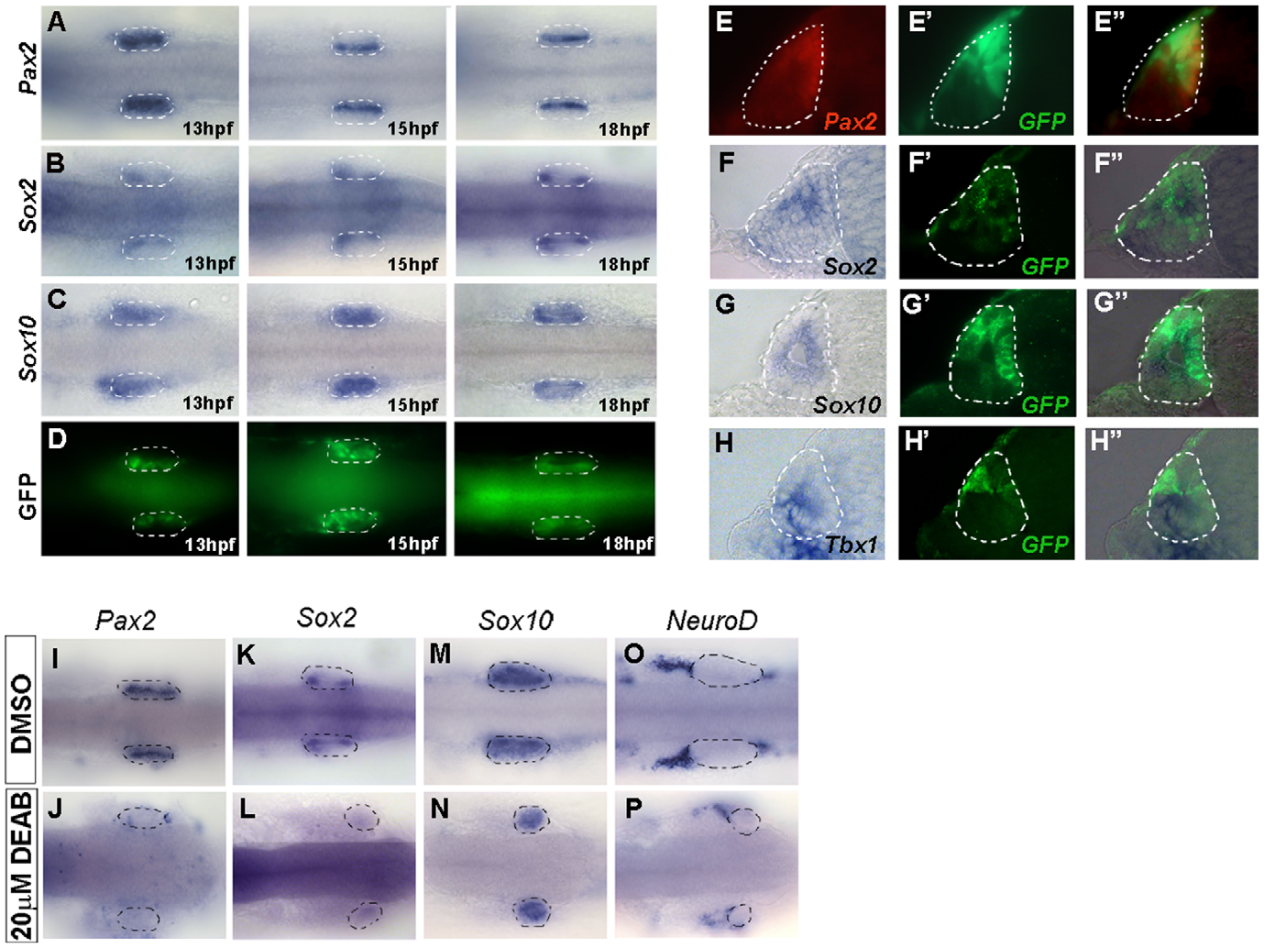
Co-localization of Pax2 and Sox2 with GFP driven by the HCNR 81675 enhancer. (A–D) Dorsal view of transgenic embryos assayed by ISH for the expression of *pax2a* (A), *sox2* (B), *sox10* (C) at 13, 15 and 18 hpf. GFP (D) displays a similar pattern than *sox2* and *pax2a* at 15 hpf (compare A and B with D). Orientation is anterior to the left. (E–E″) Double immunostaining with anti-Pax2 (E) and anti-GFP antibody (E′) in transverse sections of 15 hpf otic vesicles revealed co-localization of both proteins (E″). (F–F″) GFP protein (F′) also co-localizes with *sox2* mRNA (F″) but not *sox10* or *tbx1* mRNA (G″ and H″). (I–P) *pax2a* and *sox2* expression is abolished in retinoic acid treated HCNR 81675 embryos (compare J and L to I and K, respectively) but not other genes such as *sox10* or *neuroD* (compare N and P to M and O, respectively). Dorsal view, orientation is anterior to the left.

To get more insight about the early events during inner ear development, we then analysed in more detail how the earliest inner ear enhancer we have described, that located at HCNR 81675, is controlled. First, we compared the expression pattern promoted by this enhancer with that of several genes regionally expressed in the otic vesicle. We performed in situ hybridization of embryos of 13, 15 and 18 hpf for *pax2a*, *sox2*, *sox10* and *tbx1* and compared the patterns with GFP protein staining in HCNR 81675 transgenic animals. GFP was found in the medial otic domain also stained for *pax2a* (Figure 5A and D). *Sox2*, although more restricted to an anterior and a posterior medial region, was also found in the same medial domain as GFP (Figure 5B and D). However, there was no correlation with the other tested genes, since *sox10* was expressed all over the otic vesicle (Figure 5C) and *tbx1* was expressed in a lateral domain (data not shown). These results were further confirmed by double labelling experiments. Double immunostaining with anti-GFP antibody and anti-Pax2a showed co-localization of both proteins in the same domain (Figure 5E–E″), while anti-GFP immunostaining after in situ hybridization for *sox2*, *sox10* and *tbx1* transcripts in 15 hpf embryos revealed only co-expression of GFP and *sox2* transcripts (Figure 5F–F″). No exact correspondence with *sox10* (Figure 5G–G″) and *tbx1* domains of expression was found. Indeed, *tbx1* and GFP expression domains were mutually exclusive (Figure 5H–H″).

As aforementioned, we found that the HCNR 81675 enhancer activity is regulated by retinoic acid. Thus, to determine whether the GFP inhibition by DEAB (retinoic acid inhibitor) observed in embryos transgenic for the HCNR 81675 enhancer, correlated with abrogation of *pax2a* and/or *sox2* gene expression, in situ hybridization for these two genes as well as for *sox10* and *neuroD* was performed after DEAB treatment. Indeed, RA inhibition by DEAB leaded to a complete loss of *pax2a* and *sox2* expression in the otic vesicle, whereas *sox10* and *neuroD* expression was similar to the DMSO-treated control embryos (Figure 5I–P). All together, these results suggest that *pax2a* and *sox2* expression regulated by RA signaling would be required for the proper cis-activation of the HCNR 81675 enhancer in the otic vesicle.

### Pax2 and Sox2 are directly recruited to the *pou3f4* HCNR 81675 DNA and are required for its function

**Figure 6:**
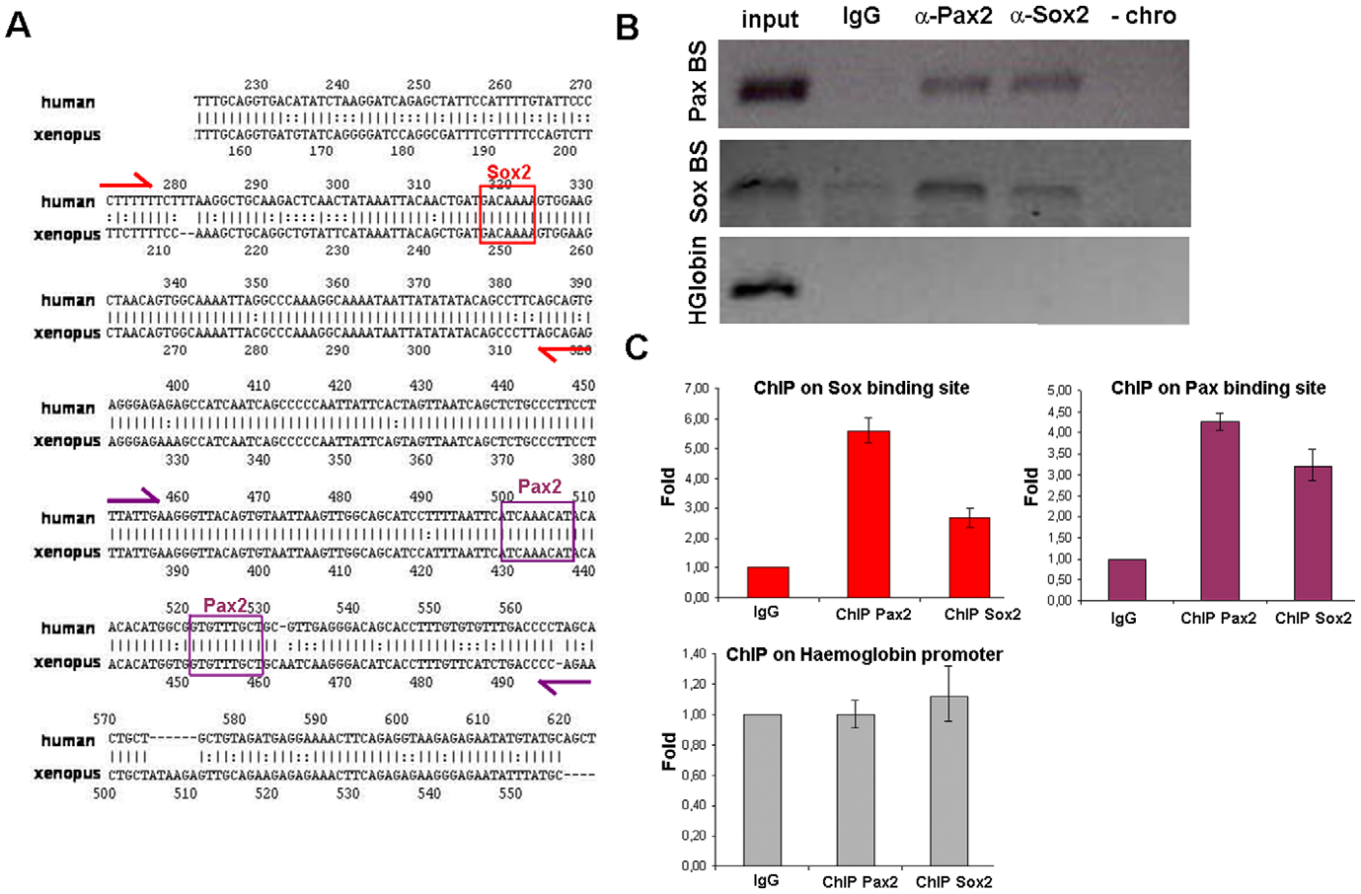
Pax2 and Sox2 are directly recruited to the HCNR 81675 DNA. (A) rVista 2.0 alignment of HCNR 81675 human and *Xenopus* genomic sequence. Conserved binding sites for Sox and Pax2/5/8 proteins found by TRANSFAC are represented. Primer location enclosing the genomic region of Sox and Pax2/5/8 binding sites designed for chromatin immunoprecipitation are marked with red and violet arrows respectively. (B) ChIP with anti-Pax2 and anti-Sox2 antibodies from stage 30–34 *Xenopus* otic vesicles was performed and the PCR amplification of the DNA fragments pulled down by Pax2 and Sox2 chromatin immunoprecipitation is shown. A region of the *haemoglobin* locus with no Pax2 and Sox2 binding sites shows no immunoprecipitation with these antibodies. (C) Graphs representing the relative fold enrichment of Sox2 and Pax2 binding to the HCNR 81675 but not to the *haemoglobin* region detected by quantitative PCR.

Since *pax2a* and *sox2* are co-expressed in a similar domain than that promoted by the HCNR 81675 enhancer and retinoic acid inhibition results in loss of both, GFP and *pax2a* and *sox2* expression, we then checked for Pax2 and Sox2 binding sites in the HCNR 81675 genomic sequence. TRANSFAC analysis of human and *Xenopus* HCNR 81675 sequences revealed a high number of conserved putative Transcription Factor Binding Sites (TFBS). Interestingly for the work presented here, a putative Sox motif and two Pax2/5/8 motifs where found in the sequence (Figure 6A). To check whether Pax2 and Sox2 proteins are directly bound to this HCNR 81675 *in vivo*, we performed chromatin immunoprecipitation assay (ChIP). PCR primers were designed to include the region of Pax2 and Sox2 binding sites. Otic vesicles from stage 30 to 34 *Xenopus* embryos were dissected and the chromatin was immunoprecipitated with either rabbit anti-Pax2 and rabbit anti-Sox2 antibodies or rabbit IgG as control. Figure 6B shows that the *pou3f4* HCNR 81675 DNA was precipitated by the anti-Pax2 and anti-Sox2 antibodies but not by the isotopic antibody. Moreover, no binding of these two proteins in the *Xenopus haemoglobin* promoter which lacks Pax2 and Sox2 binding sites was detected, confirming the specificity of Pax2 and Sox2 binding to the endogenous *Xenopus* HCNR 81675 (Figure 6B and quantitative PCR results shown in Figure 6C).

**Figure 7:**
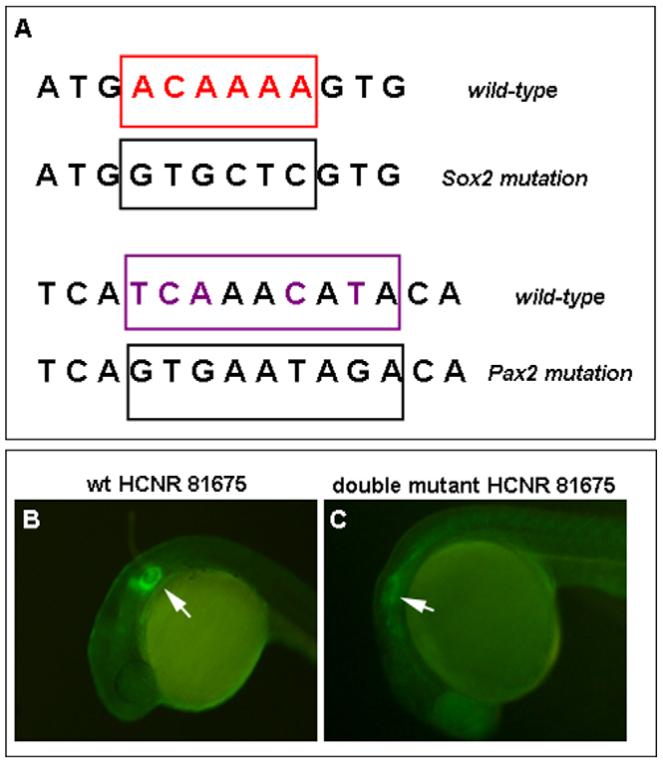
Pax2 and Sox2 proteins are required for HCNR 81675 enhancer activation. (A) Scheme showing wild-type Sox and Pax2/5/8 consensus in the *pou3f4* HCNR 81675 sequence and above each one, the mutation in the primers designed for site-directed mutagenesis of the Sox and Pax2/5/8 binding sites. (B–C) Transgenic embryos carrying GFP under the control of the HCNR 81675 enhancer. (B) GFP expression promoted by the wild type HCNR 81675 sequence. (C) GFP expression promoted by the HCNR 81675 enhancer harbouring the double mutation for Pax2/5/8 and Sox binding sites.

Finally, to further confirm that Pax2 and Sox2 proteins are required for the *in vivo* functionality of the HCNR 81675 enhancer, we designed primers containing mutations in the core nucleotides of the Sox2 and Pax2 binding sites (Figure 7A) and we performed site-directed mutagenesis of these sites in the HCNR 81675 sequence. Stable zebrafish transgenic lines were generated with a construct containing the GFP gene under the control of the *pou3f4* HCNR 81675 enhancer harbouring either the mutation for the Pax2 or Sox2 binding sites as well as double mutants. As shown in Figure 7B and 7C, GFP was dramatically reduced when both the Pax2 and Sox2 binding sites are mutated, but not in the Pax2 or Sox2 single mutants (data not shown), indicating that both proteins are directly bound to the *pou3f4* HCNR 81675 DNA region and required for its enhancer activity.

## Discussion

Hearing impairment is one of the most prevalent sensorineural defects in humans and in the last years many human ear disorders have been linked to mutations in over a hundred different genes [32], [33]. One of the most frequent causes of X-linked hereditary deafness is caused by mutations in the *POU3F4* locus. It has been described that mutations leading to X-linked deafness type 3 (DFN3) syndrome not only affect the *POU3F4* coding sequence [16] but also upstream non-coding regions, since many human patients displaying sensorineural hearing loss contain microdeletions in a region 1 Mb 5′ upstream of the *POU3F4* gene. Accordingly, we and others have recently identified several *pou3f4* inner ear enhancers within this genomic region [17], [20]. In this work, we show that these enhancers have distinct spatio-temporal activities and are activated by different signaling pathways.

### Several *pou3f4* enhancers drive otic vesicle expression

Previous work published by Ahn and colleagues described a human HCNR that specifically directs *POU3F4* expression mainly to the periotic mesenchyme in transgenic lacZ mouse embryos [17]. By screening for HCNR over a region of 1 Mb 5′ upstream of th*e POU3F4 c*oding unit several other evolutionary conserved sequences (from human to *Xenopus*) were identified [20]. In transgenic zebrafish, *Xenopus* HCNR 81675 and HCNR 82478 specifically drive reporter GFP expression to the otic epithelium and at later stages to the inner ear sensory patches. HCNR 81675 directs reporter gene expression only to the otic vesicle, while HCNR 82478 acts as a general enhancer driving *pou3f4 ex*pression to all the tissues where it is expressed such as the otic vesicle, midbrain-hindbrain boundary, mesonephros and spinal cord. Both new otic enhancers are at each side of the previously identified *POU3F4* enhancer [17]. Thus, three different enhancers drive *pou3f4* expression to the otic territory indicating that Pou3f4 is a crucial transcription factor for inner ear development and that its expression needs very fine-tuned regulation. This is further exemplified by its distinct temporal activity. In stable transgenic fishes, GFP in the otic vesicle is switched on at different developmental stages, being the enhancer at HCNR 81675 active before the one at HCNR 82478, and this one before the regulatory element located in HCNR 81728. Moreover, transcriptional activation directed by the HCNR 82478 enhancer starts in the mesonephros, followed by activity in the midbrain-hindbrain boundary and finally to the otic vesicle. Later on, at 24–32 hpf GFP is no longer detected in the mesonephros and in contrast it is activated in the spinal cord.

It has been well reported that developmental genes are very tightly regulated and thus in their loci several scattered enhancers are contained around or in the gene that regulate. Examples are found in the locus of the *Hox* and *Irx* gene clusters or the *Sox2* gene [34], [35]. In the case of *Sox2*, Kondoh and colleagues have nicely dissected out the genomic regulatory apparatus of the chicken *Sox2* locus. Eleven enhancers were identified and from those, three and two enhancers control the expression of *Sox2* in the spinal cord and otic placode, respectively.

### Different regulation and temporal activation of both otic epithelium enhancers

Inner ear development is highly complex and many distinct signaling pathways regulate it. Fgf signaling for example is used throughout ear development to control distinct processes, initially is essential for the induction of the otic primordium from the ectoderm. In zebrafish embryos, loss of *fgf3* and *fgf8* results in complete ablation of the otic placode, while in chick and mice Fgfs from the mesoendoderm primarily control the process [26]–[29], [36]–[38]. Later on, different Fgfs participate in inner ear neurogenesis, growth and morphogenesis [39]–[43]. Blockade of Fgf signaling by SU5402 did affect the size of the otic vesicles of both enhancer transgenic fish due to its role in otic growth and placode formation. GFP expression driven by HCNR 82478 but not by HCNR 81675 was suppressed by the pharmacological inhibition of the Fgf pathway at late gastrula stages. The activation of HCNR 82478 by Fgf signaling probably reflects a late role of Fgf in sensory development as several Fgfs are expressed in sensory patches in higher vertebrates [39], [43]. In contrast, the activity HCNR 81675 required the integrity of RA pathway at early gastrula stages but not at later stages. RA is synthesized in the paraxial mesoderm and influences hindbrain and ear development [44]–[46]. In zebrafish, treatment of embryos with low levels of RA causes expansion of the otic field, suggesting that RA has a role in limiting the field to respond to otic inducing signals [23]. Moreover, RA has also a positive action in the regeneration and generation of hair-cells [47], [48]. RA in addition to regulate otic development directly, at early gastrula stages is necessary for proper hindbrain patterning and establishment of hindbrain *fgf* expression [23], [26]. Thus, the role of RA on the activity over the HCNR 81675 might be indirect through the disruption of hindbrain signals and the synergistic effect of inhibiting RA and Fgf pathway at 5.5 hpf. At 9.5 hpf, both pathways are independent, in agreement with a independent regulation of the HCNR 82478 enhancer by Fgf but not RA pathway. Notch pathway has a crucial role in the specification of the sensory domains in several vertebrate species [49], [50], while BMP4 regulates the generation of the hair-cells in the sensory patches [51], [52]. For this reason, was surprising that none of the enhancers was affected by Notch or BMP inhibition. Interestingly, the late enhancer at HCNR 81728 is regulated by Hh. This would be in agreement with previous findings in which was shown that Shh signaling secreted by the notochord and/or floor plate is required for the specification of the cochlea [53]. Several data suggest that the newly found regulation of Hh over the HCNR 81728 enhancer might be mediated by Tbx1 transcription factor: first, we show a regulation of Hh over the human *POU3F4* mesenchymal enhancer; second, previous reports in mice indicated that Pou3f4 cooperates with Tbx1 during cochlear development [30]; third, *Pou3f4* expression is reduced in conditional Tbx1 mutants [54] and, finally Tbx1 mesenchymal expression is regulated by Shh [31]. Thus, here we provide for the first time an overall analysis of the signaling pathways that impact on *pou3f4* expression, acting separately over distinct enhancers located at different HCNRs.

### HCNR 81675 enhancer activity requires Pax2 and Sox2

GFP driven by *pou3f4* HCNR 81675 co-localized with the *pax2a* and *sox2* expression domains in the otic vesicle. Since Sox and Pax but not Tbx binding sites were found in the HCNR 81675 sequence, we hypothesized that both Pax2 and Sox2 might be activating the enhancer in the zebrafish transgenic line. This was confirmed by chromatin IP and site-directed mutagenesis of the Pax2 and Sox2 binding sites that showed that Pax2 and Sox2 TF are bound directly to the enhancer and regulate GFP expression *in vivo*. Altogether, our results suggest that early *pou3f4* expression directed by the HCNR 81675 enhancer may be regulated by retinoic acid and Sox2 and Pax2 transcription factors. Our mutagenesis analysis of the HCNR 81675 enhancer plus the fact that GFP is only found in domains of pax2a and sox2 co-expression, indicate that Pax2 and Sox2 transcription factors alone are not sufficient to activate this enhancer and act in a cooperative manner over the genomic locus. This would be similar to what has been reported by the cooperative interaction of Pax6 and Sox2 in the δ*-crystallin* and N3 *Sox2* enhancers [55].

Endogenous *pou3f4/Pou3f4* gene is expressed in the midbrain-hindbrain boundary, mesonephros and spinal cord, as well as the periotic mesenchyme in *Xenopus* and mouse embryos [13] (Figure S4). Several possibilities might explain the difference between the endogenous *pou3f4* expression in the periotic mesenchyme and the activation of the *pou3f4* enhancers in the otic epithelium in zebrafish. First, endogenous *pou3f4* transcripts might be present in the otic epithelium at lower and undetectable levels by in situ hybridization; secondly developmental differences of expression might appear when enhancers are extracted from their genomic context. The latter hypothesis of an improper regulation of foreign enhancers is favoured by the fact the human enhancer described by Ahn et al. 2009 when inserted in mice [17] drives ectopic lacZ expression in the spiral ganglion in addition to the mesenchymal expression and in zebrafish (our manuscript and [20]) expression is mainly found in the otic vesicle. Moreover, it has been recently hypothesized that fine-tune gene expression required during embryogenesis would be the result of the synergistically interaction of different enhancer elements in a combinational manner [56]. Following this notion, large genomic regions containing several regulatory elements have under-representation of nucleosomes suggesting a higher-order genomic structure [57], [58].

In conclusion, the description of the spatiotemporal activity of novel enhancers of *POU3F4* gene and their regulation may contribute to the further understanding of the function of POU3F4 in inner ear and its implications in DNF3.

## Supporting Information

Figure S1 **HCNR 81675 activity is not dependent on RA, Fgf, Notch, Bmp and Hh at 7.5 and 9.5 hpf.** (A–N) GFP is observed after treatment of HCRN 81675 transgenic embryos with pharmacological inhibitors of signaling pathways at 7.5 hpf (A–G) and 9.5 hpf (H–N). Orientation is anterior to the left and dorsal up.
(TIF)

Figure S2 **Abrogation of different signaling target genes after treatment with specific signaling inhibitors.** (A–J) In situ hybridization for the Fgf, Notch, Retinoic Acid, BMP and Sonic Hedgehog target genes *pea3* (A–B), *neuroD* (C–D), *krox20* (E–F), *msxC* (G–H) and *ptc1* (I–J) to confirm inhibitor activity at our working concentrations. Dorsal view, orientation is anterior to the left.
(TIF)

Figure S3 **HCNR 82478 activity is dependent on Fgf signaling when treated at 5.5 and 7.5 hpf.** (A–N) GFP is inhibited after treatment of HCRN 82478 transgenic embryos with 30 µM SU5402 at 5.5 hpf (A–G) and 50 µM SU5402 at 7.5 hpf (H–N). Orientation is anterior to the left and dorsal up in all images.
(TIF)

Figure S4 **Endogenous expression pattern of** ***pou3f4/Pou3f4*** **in** ***Xenopus*** **and mouse**. (A–B) In situ hybridization for *pou3f4* mRNA in *Xenopus* embryos of stage 35 (A) and stage 42 (B). Note that the endogenous expression is detected at the periotic mesenchyme at stage 42, whereas at stage 35 *pou3f4* is still not expressed. (C–D″) In situ hybridization for *Pou3f4* mouse mRNA in mice embryos of stage E8.5 (C) and E16.5 (D). In mice, also *Pou3f4* is expressed at the otic mesenchyme at later stages, shown in insets (D′, D″). Transverse sections shown in all panels.
(TIF)
