# Supplementary figures and images for: Characterization of New Otic Enhancers of the Pou3f4 Gene Reveal Distinct Signaling Pathway Regulation and Spatio-Temporal Patterns
Source: PLoS One. 2010 Dec 31;5(12):e15907. doi: 10.1371/journal.pone.0015907 (PMC3013142; doi:10.1371/journal.pone.0015907)

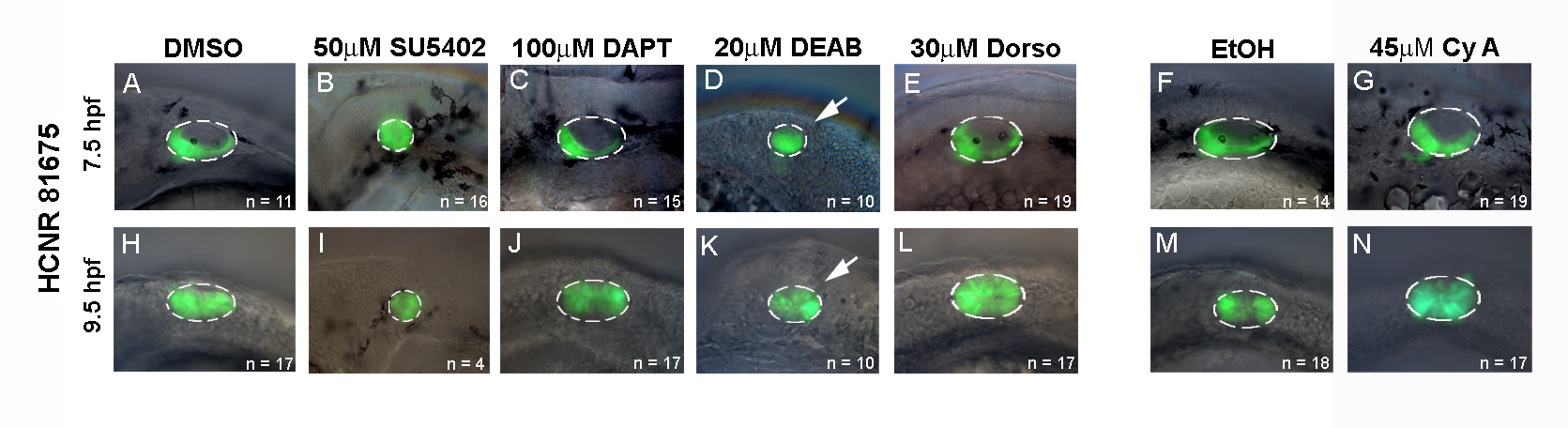

Supplement: Figure S1 — HCNR 81675 activity is not dependent on RA, Fgf, Notch, Bmp and Hh at 7.5 and 9.5 hpf. (A–N) GFP is observed after treatment of HCRN 81675 transgenic embryos with pharmacological inhibitors of signaling pathways at 7.5 hpf (A–G) and 9.5 hpf (H–N). Orientation is anterior to the left and dorsal up. (TIF) [file pone.0015907.s001.tif]

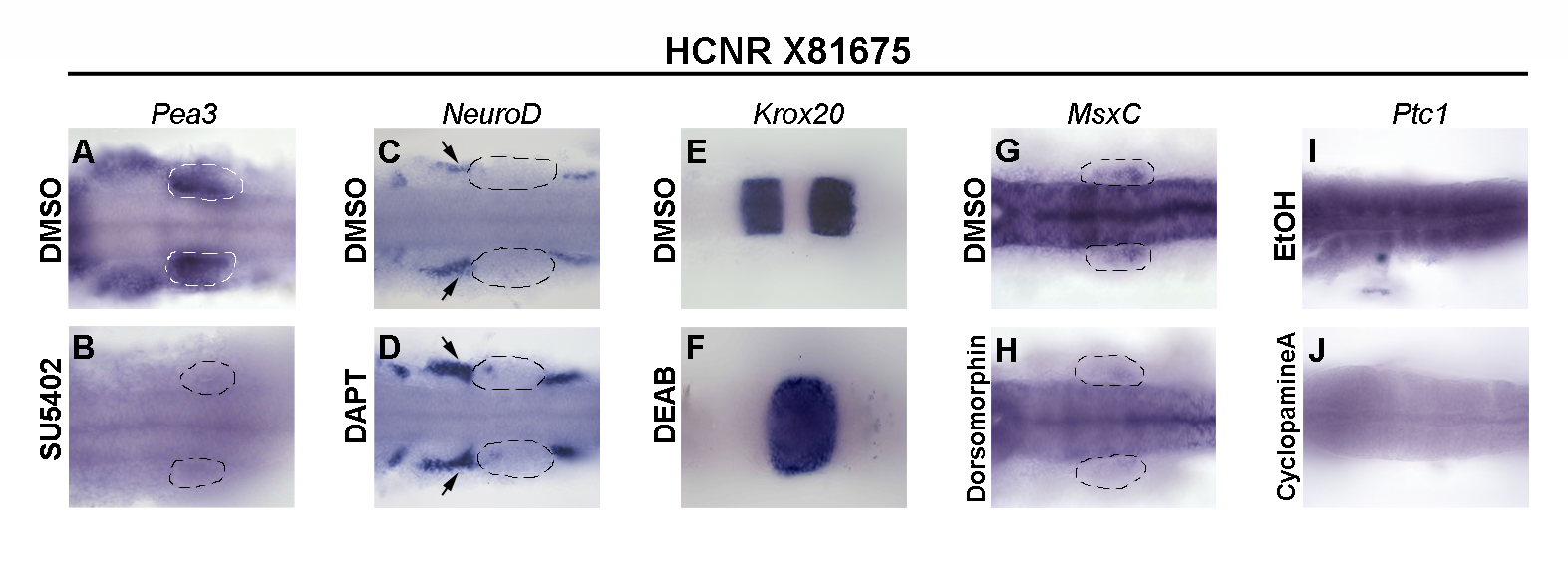

Supplement: Figure S2 — Abrogation of different signaling target genes after treatment with specific signaling inhibitors. (A–J) In situ hybridization for the Fgf, Notch, Retinoic Acid, BMP and Sonic Hedgehog target genes pea3 (A–B), neuroD (C–D), krox20 (E–F), msxC (G–H) and ptc1 (I–J) to confirm inhibitor activity at our working concentrations. Dorsal view, orientation is anterior to the left. (TIF) [file pone.0015907.s002.tif]

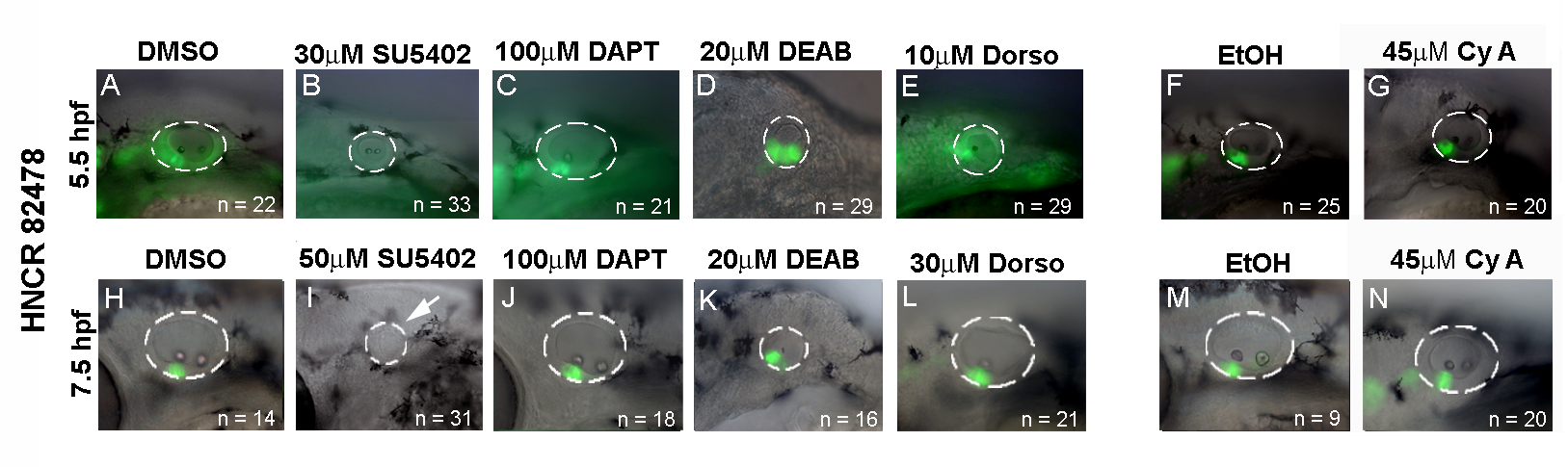

Supplement: Figure S3 — HCNR 82478 activity is dependent on Fgf signaling when treated at 5.5 and 7.5 hpf. (A–N) GFP is inhibited after treatment of HCRN 82478 transgenic embryos with 30 µM SU5402 at 5.5 hpf (A–G) and 50 µM SU5402 at 7.5 hpf (H–N). Orientation is anterior to the left and dorsal up in all images. (TIF) [file pone.0015907.s003.tif]

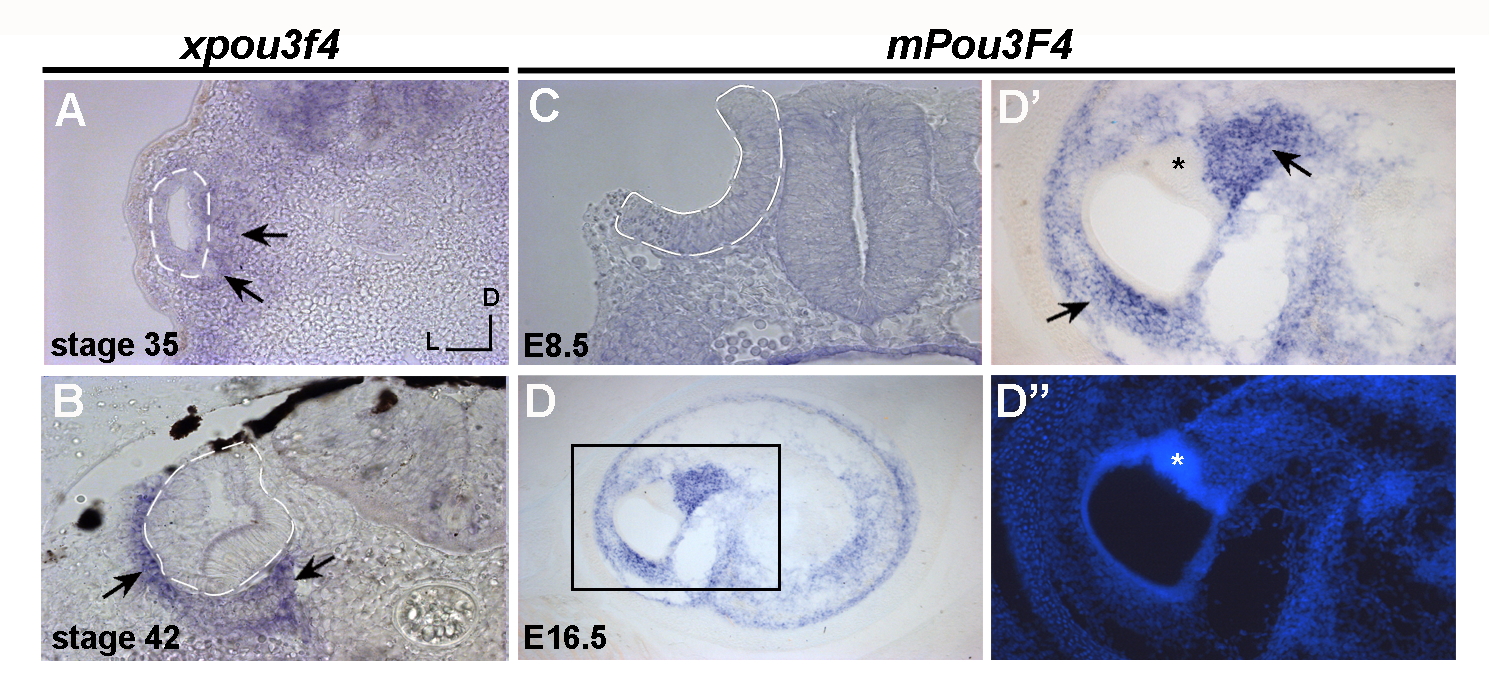

Supplement: Figure S4 — Endogenous expression pattern of pou3f4/Pou3f4 in Xenopus and mouse. (A–B) In situ hybridization for pou3f4 mRNA in Xenopus embryos of stage 35 (A) and stage 42 (B). Note that the endogenous expression is detected at the periotic mesenchyme at stage 42, whereas at stage 35 pou3f4 is still not expressed. (C–D″) In situ hybridization for Pou3f4 mouse mRNA in mice embryos of stage E8.5 (C) and E16.5 (D). In mice, also Pou3f4 is expressed at the otic mesenchyme at later stages, shown in insets (D′, D″). Transverse sections shown in all panels. (TIF) [file pone.0015907.s004.tif]
